# Erratum for Poonsin et al., “Canine respiratory coronavirus in Thailand undergoes mutation and evidences a potential putative parent for genetic recombination”

**DOI:** 10.1128/spectrum.01139-26

**Published:** 2026-07-10

**Authors:** Panida Poonsin, Vorapun Wiwatvisawakorn, Jira Chansaenroj, Yong Poovorawan, Chutchai Piewbang, Somporn Techangamsuwan

## ERRATUM

Volume 11, no. 5, e02268-23, 2023, https://doi.org/10.1128/spectrum.02268-23. Acknowledgments: “P.P. received a grant from The Thailand Research Fund through the Royal Golden Jubilee Ph.D. Program (Grant No. NRCT5-RGJ63001-013) and The Second Century Fund (C2F), Chulalongkorn University” should read “This research project is supported by National Research Council of Thailand (NRCT): [NRCT5-RGJ63001-013] and The Second Century Fund (C2F), Chulalongkorn University (to P.P.).”

