# Supplementary material for: Canine respiratory coronavirus in Thailand undergoes mutation and evidences a potential putative parent for genetic recombination
Source: Microbiol Spectr. 2023 Sep 14;11(5):e02268-23. doi: 10.1128/spectrum.02268-23 (PMC10581155; doi:10.1128/spectrum.02268-23)
Supplement: Tables S1 to S2, Fig. S1 — Primer list, selective pressure analysis, SimPlot analysis. [file spectrum.02268-23-s0001.docx]

**TABLE S1** Primers used in this study.

| Primer | Sequence (5’-3’) | Position | Product size (bp) | Annealing temperature (°C) |
| --- | --- | --- | --- | --- |
| Orfab-1F | CTATGCTTGTGGGCGTAGATTT | 31-52 | 1342 | 55 |
| Orfab-1R | CCATCTATCATATTACCAGGAACC | 1372-1349 |  |  |
| Orfab-2F | CTTGTGTGATGGTTCTGTTGTTAT | 1127-1150 | 1425 | 55 |
| Orfab-2R | CATGGTGTAAGTGAAGTTGTAAC | 2551-2529 |  |  |
| Orfab-3F | GTTGAGCGTGGCTTGTTACATTC | 2379-2401 | 1424 | 56 |
| Orfab-3R | TCCACAACACAAGCTGCTTTATAC | 3802-3779 |  |  |
| Orfab-4F | CACAAGGTGGTTATGTTGCTGAT | 3517-3539 | 1134 | 58 |
| Orfab-4R | CATCAACATTGCTCTGAACAAAGG | 4650-4627 |  |  |
| Orfab-5F | GTGTCAGATAACTGCCGTTGAGG | 4430-4452 | 1276 | 60 |
| Orfab-5R | GCCAGTAACGTCTGTAACCTTC | 5705-5684 |  |  |
| Orfab-6F | GCATTTTGGTACATTGAGTCGTG | 5438-5460 | 1531 | 58 |
| Orfab-6R | GCAGAACTGACATGCGATACTTC | 6968-6946 |  |  |
| Orfab-7F | GGCTGGATTAAAATATCCGCTG | 6603-6624 | 1612 | 58 |
| Orfab-7R | CAACATTACCCTGCACATGCTTAG | 8214-8191 |  |  |
| Orfab-8F | CTGCTAATACTGGTACGTCTGTTA | 7837-7860 | 1573 | 55 |
| Orfab-8R | CAAGAAAGTGTAGGGTAAACTTG | 9409-9387 |  |  |
| Orfab-9F | GATCATTGCCTGGGACCTTTTG | 9136-9157 | 1339 | 60 |
| Orfab-9R | TAATCCTGAACGGGCAATTGGAC | 10474-10452 |  |  |

**TABLE S1** Primers used in this study (continued).

| Primer | Sequence (5’-3’) | Position | Product size (bp) | Annealing temperature (°C) |
| --- | --- | --- | --- | --- |
| Orfab-10F | GAGCCTTTCATGTGACTATGCGTA | 10252-10275 | 1118 | 58 |
| Orfab-10R | CACAGCAACCCACTTAGCAATAAC | 11369-11345 |  |  |
| Orfab-11F | CACTGCTGTATAACAACTATTTGG | 11017-11040 | 892 | 54 |
| Orfab-11R | GTATTGTCCTTTGCGTAATCGTC | 11908-11886 |  |  |
| Orfab-12F | GCTAATGGATTGCGCCCTCCTAAG | 11547-11570 | 1410 | 60 |
| Orfab-12R | GGCAATAGGTGTTCCTCCCTGTTG | 12956-12933 |  |  |
| Orfab-13F | GCTATACTTAGTGATGTTGATGG | 12630-12652 | 1485 | 55 |
| Orfab-13R | GCATACTCCAGTGCTTAAAATAC | 14114-14092 |  |  |
| Orfab-14F | GGTAAATGGTATGATTTTGGTGAC | 13889-13912 | 1519 | 55 |
| Orfab-14R | GAAGAGCACGTATACTCAAATC | 15407-15386 |  |  |
| Orfab-15F | CCTAAGTGTGATCGTGCTATGCC | 15095-15117 | 1463 | 60 |
| Orfab-15R | GGTGGCTTCACTTTTCCAATCTCC | 16557-16534 |  |  |
| Orfab-16F | GTACAGGATCTCCGTACATAGATG | 16323-16346 | 1269 | 55 |
| Orfab-16R | CAAAGTTCTGACTATTATATGGGC | 17591-17568 |  |  |
| Orfab-17F | GTTATTGAGCAAGGGTACACTTG | 17260-17282 | 1422 | 55 |
| Orfab-17R | GCCAAACACCGTGTCATTATAGC | 18681-18659 |  |  |

**TABLE S1** Primers used in this study (continued).

| Primer | Sequence (5’-3’) | Position | Product size (bp) | Annealing temperature (°C) |
| --- | --- | --- | --- | --- |
| Orfab-18F | GCCACTGGTTTGTTTGCTGATAG | 18200-18222 | 1585 | 60 |
| Orfab-18R | CTTCAAGAGCACCATTATCACGA | 19784-19762 |  |  |
| Orfab-19F | GCAGTATTCGACACCATCCAGAG | 19587-19609 | 1055 | 60 |
| Orfab-19R | TGTAGGCAAAGTAACTGGCTTCC | 20641-20619 |  |  |
| Orfab-20F | GTTTCATACGACTCCAGCATACAC | 20294-20317 | 1314 | 55 |
| Orfab-20R | CTGAATATCCAGCATAGCAAGAC | 21607-21585 |  |  |
| 32kDa NS F | GCTAAATTCCCGCTTAAGTT | 21269-21288 | 1147 | 56 |
| 32kDa NS R | CAATCTGAACGACTGTCACC | 22415-22396 |  |  |
| ^a^HE1F | CATCACCGGCTAGACTTGAA | 21924-21943 | 1258 | 59 |
| ^a^HE1R | AGACTGCCTGGCATTGTTCC | 23181-23162 |  |  |
| HE2F | CTCTGCACAATCTACAGCTC | 22746-22765 | 880 | 60 |
| HE2R | CTCCTATAACAGCAAAAGCCATTG | 23625-23602 |  |  |
| Spike-1F | TCGTAATATGGCACTGAAGGGAA | 23544-23563 | 1374 | 60 |
| Spike-1R | CCAAATAGAAGGATTAAACCTGC | 24917-24895 |  |  |
| Spike-2F | GATAAGTTTGCTATACCCAATGG | 24750-24772 | 1082 | 59 |
| Spike-2R | CCTACTGTGAGATCACATGTTTG | 25831-25809 |  |  |

**TABLE S1** Primers used in this study (continued).

| Primer | Sequence (5’-3’) | Position | Product size (bp) | Annealing temperature (°C) |
| --- | --- | --- | --- | --- |
| Spike-3F | CTTGGTTGTGTTGTCAATGC | 25764-25783 | 1453 | 58 |
| Spike-3R | CATTATTTCCAGTTATAGGTTCAGG | 27216-27192 |  |  |
| ^b^Spike-4F | ACACTTGGATGTTCACTGGTAG | 27157-27178 | 351 | 58 |
| ^b^Spike-4R | GCCATACATACCAAGGCCATTTTA | 27507-27484 |  |  |
| Spike-5F | GGACCTACAAGATGAAATGAATAG | 27386-27409 | 952 | 60 |
| Spike-5R | CAGGTAATAAAGTCAACAGCTAAG | 28337-28314 |  |  |
| G1F | GTATGTATGGCTTTTAATTGGC | 27497-27518 | 1105 | 57 |
| G1R | CATCCACATCAAGAACTGGTG | 28601-28581 |  |  |
| M1F | CAGACACTGTGTGGTATGTGG | 28380-28400 | 1089 | 60 |
| M1R | GTTTGCTTGGGTTGAGCTCTTC | 29468-29447 |  |  |
| N1F | CAGTAGTAGAGCGTCCTCTGG | 29352-29372 | 1517 | 56 |
| N1R | GGCAATTACTTCCGCAAGTC | 30868-30849 |  |  |

The oligonucleotide position is based on the sequence of CRCoV strain BJ232 (KX432213.1).

^a^HE1F-1R is the primer from previously published (An et al., 2010).

^b^Spike4F-4R is the primer for CRCoV RT-PCR screening test.

**TABLE S2** Selective pressure analysis using various detection methods.

| CRCoV gene | Selective pressure analysis | | MEME | SLAC | FEL | FUBAR |
| --- | --- | --- | --- | --- | --- | --- |
| Spike | Positive selection | 2 | | 0 | 1 | 6 |
|  | Negative selection | - | | 16 | 52 | 45 |
|  | Overall dN/dS | 0.326 | | 0.362 | N/A | N/A |

**
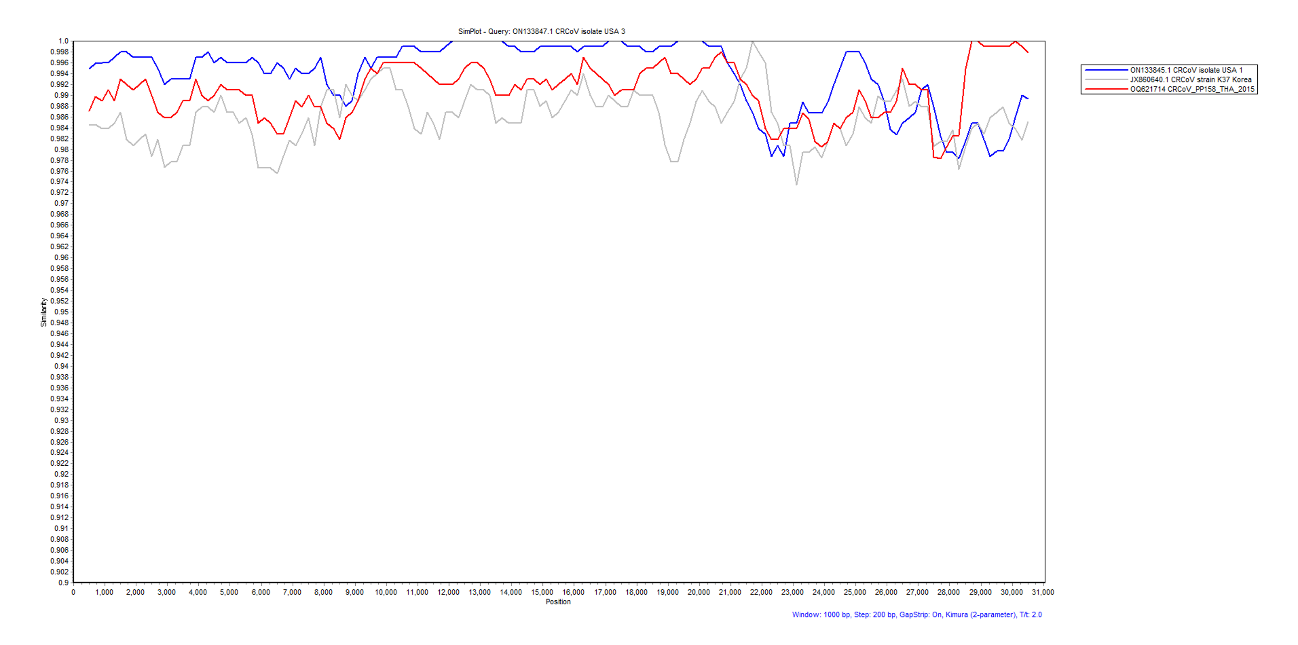
FIG S1 The results of a SimPlot similarity analysis.** The recombinant CRCoV isolate USA 3 (ON133847.1) was used as a query sequence and compared with CRCoV isolate PP158_THA_2015 (OQ621714; red line), CRCoV isolate USA 1 (ON133845.1; blue line), and CRCoV strain K37 Korea (JX860640.1; gray line). The y-axis indicates the nucleotide similarity. A window size of 1,000 bp and step size of 200 bp were used for SimPlot analysis.

**Reference**

1. An D-J, Jeong W, Yoon SH, Jeoung H-Y, Kim H-J, Park B-K. 2010. Genetic analysis of canine group 2 coronavirus in Korean dogs. Vet Microbiol 141:46-52
